# *Sakshi* and *Dhyana*: the origin of mindfulness-based therapies

**DOI:** 10.1192/bjb.2022.39

**Published:** 2023-04

**Authors:** Swaran P. Singh

**Affiliations:** Warwick Medical School, Coventry, UK; University of Warwick, Coventry, UK; Coventry and Warwickshire NHS Partnership Trust, Coventry, UK

**Keywords:** Mindfulness, psychotherapy, transcultural psychiatry, philosophy, spirituality

## Abstract

Mindfulness-based therapies (MBTs) have shown promising results in non-psychotic disorders. Unlike most other psychotherapy models, which are claimed to be Western in origin, MBTs are firmly based in Indian philosophy and traditions. This paper summarises the concepts of the observer self (*sakshi*) and attention (*dhyana*) that underlie the principles and practice of MBT, correcting some erroneous assumptions in the process. It is argued that better understanding of these concepts is beneficial not just for specialist psychotherapists, but for all clinicians interested in the craft of healing.

Western scholarship traces the origins of philosophy, mathematics, science and rationality to the ancient Greeks. Books and articles on philosophy often open with statements such as ‘if philosophy has a patron saint, it is Socrates’.^1^ Plato is considered ‘the first person to propose a theory of knowledge and he, or Socrates, first proposed using reason to decide moral questions’.^2^ Bertrand Russell, in his famous *History of Western Philosophy*, claims: ‘they [the Greeks] invented mathematics, science and philosophy’.^3^ Thomas Macaulay (1800–1859), a Whig politician who served on the Supreme Council of India, famously claimed that ‘a single shelf of a good European library [is] worth the whole native literature of India and Arabia’.^4^

In today's information-rich world, it takes a cursory internet search to refute these claims. Thinkers from India, China and other non-European regions were asking and debating questions about morality, existence, virtue and afterlife well before the Hellenic period considered the birth of philosophy. If one purpose of history is to teach human beings not to repeat mistakes, historians and academics have singularly failed in correcting the erroneous claims about the supposed Western origins of many contemporary disciplines. One such discipline, the study of the mind (encompassing much of what we now term psychology), is also supposed to have begun with the Greeks. Ancient Hindu, Buddhist and Jain philosophies have sophisticated discourse and debates on the nature of mental phenomena, epistemology and suffering that pre-date anything similar in the Western world.^5^ In recent years, however, one form of psychological intervention has begun to trace its origins to its ancient Indian roots: mindfulness-based therapies (MBTs).

In this paper I briefly present the historical concepts underlying MBTs and situate these in a pan-Indian understanding of mind, self, suffering and awareness. I argue that these ancient Indian concepts contain fundamental truths with applicability to most contemporary psychotherapies. I also argue that, rather than focusing on narrow differentiation between distinct schools, an overall understanding of these concepts can help a clinician use these in an eclectic manner, without needing to be trained in any highly specialised model. An interested reader offering such therapies, personally practising meditation or simply curious should gain a better understanding of the roots of mindfulness. I also hope to pique the interest of some psychiatrists in the profound, deep and rich schools of Indian philosophy, particularly phenomenology as it pertains to self and suffering.

Details of the relationship between Buddhism and its influence on other Indian traditions is beyond the scope of this paper. Briefly, Buddhism arose within a pre-existing Hindu (or more correctly Vedic, since the term Hindu was a later invention) culture. Siddhartha Gautama (The Buddha) was born to a Hindu family. As Buddhism spread, many of its concepts became absorbed into Hinduism, with some Hindus even believing that Buddha was an *avataar* – a reincarnation of a Hindu God). Buddhist doctrine is considered among the six main schools of Indian philosophy – *darshana*.^6^ A key difference between Hinduism and Buddhism is that the latter, like Jainism and Sikhism, does not accept the primacy of the Vedas or Hinduism's emphasis on rituals or the caste system. Mainstream Buddhist philosophy is, however, ‘in line with previous Hindu thought and the philosophy of the Vedanta (Upanishads)’.^7^

For readers interested in the philosophical exchanges between Ancient Greece and India, one of the best resources is *The Shape of Ancient Thought* by Thomas McEvilley;^8^ and for the wider overlap between diverse traditions, *Eastern Religions and Western Thought* by Sarvepalli Radhakrishnan is highly recommended.^9^

## Third-wave therapies

Mindfulness-based therapies, which succeeded behavioural therapies and cognitive–behavioural therapy (CBT), are considered a third wave.^10^ It is usually claimed that ‘Buddhist traditions first explored the concept of mindfulness in broad philosophical terms’.^11^ The history of MBT and its derivatives is well-presented by Geoffrey Samuel, who considers the mindfulness movement to have become ‘a significant force within contemporary society’.^12^ Interest in mindfulness is attributed to Jon Kabat-Zinn, who in 1979 introduced the mindfulness-based stress reduction (MBSR) programme at the University of Massachusetts Medical Centre to manage chronic pain and stress-related disorders.^13^ The roots of MBT are thought to be within Theravada Buddhism although, as Samuel notes, much of what passes for contemporary Western Buddhism is a ‘modernist project’,^12^ often far removed from the original Buddhist philosophy. The Buddha believed that all phenomena were transient, illusory and of dependent origination, including the self,^5^ which is fundamentally at odds with the intense psychotherapeutic focus on the self as an entity.

Overall, systematic reviews and meta-analyses show that MBTs, including mindfulness-based cognitive therapy, dialectical behaviour therapy, acceptance and commitment therapy, brief mindfulness interventions, and smartphone and internet-based interventions are superior to waiting-list controls and as effective as traditional CBT in a range of anxiety and depressive disorders, with promising results emerging in pain disorders, addictions and post-traumatic stress disorder.^11,14–16^

In MBT interventions, therapists train patients to pay attention to present-moment experience (awareness), usually with a focus on breathing, while dispassionately observing distracting mental phenomena (‘this is an angry thought’, ‘this is discomfort in the physical body’, etc.). Monitoring instructions differ across different MBT programmes, with some simply observing and others labelling the present-moment experience, but all involve a focused observation of mental activity. In some forms of mindfulness training, a chosen image (for instance a flame) or a *mantra* (chant) is used to train attention.

The second key feature of MBT is training patients to develop an attitude of acceptance and non-reactivity to momentary experience. A non-judgemental stance, with gentle and patient curiosity, even for unpleasant and distressing experiences, is encouraged, and mental distress is observed with kindness and compassion. Several mechanisms have been postulated to explain the effect of MBTs – acceptance, decentring (a third-person observation of internal phenomena), better emotion regulation, reduction in ruminations and change in one's self concept – and continue to be researched.^15,16^

## The origins of mindfulness

Mindfulness is a major feature of all Indian religious and philosophical traditions, as described below. Many of the Indian (Sanskrit) terms in this paper do not have a precise English equivalent. Almost all have complex and overlapping meanings and are used slightly differently by different schools. For the sake of brevity, I have used the closest English word or phrase.

### *Res cogitans* and *Sakshi*

The 17th-century French philosopher Descartes, using a process of doubting his perceptions and beliefs, stated: ‘after considering everything very thoroughly, I must finally conclude that this proposition, *I am*, *I exist*, is necessarily true whenever it is put forward by me or conceived in my mind’.^17^ Descartes could not doubt his doubting, hence *cogito ergo sum* – I think therefore I am. *Res cogitans* was the thinking thing whose existence he could not doubt; the rest was *res extensa* (the extended thing). Several millennia before Descartes, Indian *rishis* (seers) had concluded something similar.^5^

In Indian philosophy, a core epistemological concept is *pramaana* (proof), since true knowledge is considered the route to liberation of the self from the cycle of existence (*samasara*) and suffering (*dukkha*). *Avidya* (lack of knowledge) is ignorance about the true nature of reality. There are six kinds of *pramaana*: *pratyaskha* – that which is directly perceived through the sense organs; *anumaan* – inference of truth from observation and application of reasoning; *upmaan* – comparison, truth by analogy; *arthapatti* – presumption, a postulate that is derived from circumstances; *anupalabdhi* – non-perception, or a negative proof; and *sabda –* literally, word that is true by testimony or doctrinal authority.^18^

A particular form of negation, *neti neti,* is salient in all main Indian traditions. *Neti neti* (not this, not this) is a method of inquiry to find out what you are not: I am not this body, I am not these emotions, I am not this thought or experience, etc. By negating everything that is ‘not-self’, one is left with only the self.^19^ Hindu's call this self *atman* (soul). *Atman* serves the function of an onlooker, *sakshi* (one who sees), also sometimes described as the witness consciousness. *Sakshibhava* (the witness mode) is a state of awareness and witnessing of one's own mind and is the beginning of the process of transcending the self. Buddhists, however, believe that the doctrine of negation leads to a realisation of *anatman* (non-self), i.e. a permanent self does not exist. An observing self observes; it does not, however, exist in any permanent form.

### *Dhyana* and meditation

*Dhyana* has multiple meanings (variously understood as awareness, attention, focus, meditation, contemplation) and depending on the meaning chosen, the date of its origin varies. Srinivasan^20^ has described the subtle and nuanced ways in which the term is used in different traditions. *Dhyana* is part of all four major Indian religions: Hinduism, Buddhism, Jainism and Sikhism. Buddhism and Jainism are known as the Sramanic religions (*sraman* means effort), since both believe in asceticism and renunciation of worldly affairs and reject the rituals and doctrinal authority of the Vedas, central to Hinduism. As Buddhism travelled out of India, it evolved into several different schools and traditions, but the concept of *dhyana* stayed central to all its forms. It became *Chan* in China and *Zen* in Japan but its central meaning of awareness remained.^21^

Meditation is central to both Buddhist and Jain traditions but not exclusive to them.^5^ Ancient Hindu texts such as the *Upanishads* and the *Bhagwad Gita* mention meditation as one form of liberation from suffering. The *Upanishads*, also known as *Vedanta* (last section, of the Vedas), are a collection of philosophical discussions and aphorisms that discuss extensively the role of *dhyana* in understanding the nature of self and reality. The *Dhyanabindu* (one-pointed) *Upanishad* is devoted to the role of *dhyana* in understanding the self (*atman*) as part of a whole (*brahman*).^5^ In Sikhism, *naam simran* (meditating on the name of God) is a salient form of worship. Sikhism rejects worldly renunciation espoused in Hinduism, Buddhism and Jainism and instead asks its followers to stay engaged in the world while remaining true to God. The Sikh holy book the *Guru Granth Sahib* describes *dhyana* as ‘*panchan ka guru ek dhyan’* (awareness is the Guru of the five senses).

### *Viveka* and *Vairagya*

Yoga (from the root *yog*-, to join) is the ancient Indian practice of physical, mental and spiritual discipline which leads to the liberation of the self from a cycle of suffering.^9^ Detailed description of Yoga and its practices is beyond the scope of this paper. Briefly, yogic practices have eight *angas* (limbs): *yama* (restraint), *niyama* (discipline), *asana* (posture), *pranayama* (breath control), *pratayahara* (withdrawal of the senses), *dharana* (attention), *dhyana* (meditation) and *samadhi* (deep absorption). In the context of mindfulness, yoga emphasises four cardinal virtues: *viveka, vairagya, shat sampatti* and *mumukshutva*. *Viveka* means discernment, the ability to distinguish between what has value and is lasting and what is ephemeral, transient and illusory. *Vairagya* means non-attachment to worldly and sensual pleasures and a dispassionate approach to events. *Shat sampatti* is one of the six virtues, which are: *shama* – tranquillity, equanimity; *dama* – ability to control the senses, non-reactivity; *uparati –* renouncing what does not fit with *dharma* (virtuous duty); *titiksha –* forbearance, persevering through suffering; *shraddha* – trust and faith in the path of virtue; and *samadhi* – total concentration, highest state of contemplation. *Mumukshutva* is an intense yearning for self-realisation. *Abhyaasa* (practice) and *sadhana* (disciplined effort) are essential for an individual to attain *samadhi*, the highest meditative state.

One can easily see parallels between MBT goals and techniques and these Indian practices. The 4th-century Indian philosopher Adi Shankracharya,^22^ who resurrected the advaita (non-dual) school of Hindu philosophy, wrote a poem called *Vivekachudamani* (*The Crest Jewel of Discernment*), many translations of which are readily available on the internet. It describes the goals and methods of self-realisation through meditation. A brief book *Sadhana: Christian Exercise in Eastern Form* by the Jesuit priest Anthony De Mello describes how the spiritual exercise of *dhyana* can assist in Christian prayer and contemplation.^23^

## Clinical utility

Psychotherapeutic models are often criticised for being ‘Western’ in both their theoretical basis and practical application, with widespread calls for their cultural adaption.^24,25^ MBTs are rare examples of a non-Western belief system and philosophy being used in the West. This is a heartening reminder of both the universality of suffering and attempts to ameliorate it: heartening since culture is often considered either too vague or too difficult an aspect of the patient's background for therapists to engage with it meaningfully.^26^

Ancient wisdoms persist through the ages because they contain eternal truths.^27^ Notwithstanding the extraordinary advances in neurosciences and the exceptional role that psychopharmacology has played in alleviating human suffering, at their innermost core our patients seek understanding and meaning of their distress, over and above remission from symptoms. This is partly the reason why psychotherapies continue to be popular and in demand across a wide range of mental health services.^28^

As the historical and cultural roots of the MBTs show, there is far more in common between different psychotherapeutic models than what makes them distinct. I believe that an improved knowledge of the cultural background of such therapies can encourage clinicians to explore these often-neglected aspects of patient–therapist interaction. As psychiatry becomes more checklist driven and symptom-remission oriented, it is important for the profession to remind itself of the value of the therapeutic relationship and helping patients make sense of their experiences. We need not all be trained in highly specialised models of psychotherapy; teaching our patients the value of acceptance, detachment and non-reactivity can be major contributors to healing, beyond an effort to ‘conquer’ symptoms. Reminding patients that the mind is distractible, especially under distress, that change is difficult and needs persistence and practice and that an observer stance to the vicissitudes of life is both wise and therapeutic are all messages with everyday clinical use and should not be the realm only of specialist psychotherapists. Psychiatrists can also benefit from fruitfully engaging with a large, deep, rich and rewarding philosophical literature above and beyond that focused only on the ancient Greek period, especially in the phenomenology of self, suffering and solace.

## Data Availability

Data availability is not applicable to this article as no new data were created or analysed in this study.

## About the author

**Swaran P. Singh** is Professor of Social and Community Psychiatry at Warwick Medical School, Director of the Centre for Mental Health and Wellbeing Research at the University of Warwick, Associate Medical Director for Research and Innovation with Coventry and Warwickshire NHS Partnership Trust and a consultant psychiatrist for the North Warwickshire early intervention in psychosis service, Coventry, UK.
